# Dietary Factors and Risk of Chronic Obstructive Pulmonary Disease: a Systemic Review and Meta-Analysis

**Published:** 2019-04

**Authors:** Ensiyeh Seyedrezazadeh, Masoud Pour Moghaddam, Khalil Ansarin, Mohammad Asghari Jafarabadi, Akbar Sharifi, Sangita Sharma, Fariba Kolahdooz

**Affiliations:** 1 Tuberculosis and Lung Disease Research Center, Tabriz University of Medical Sciences, Tabriz, Iran; 2 Mt Kuring-Gai Medical Centre, Suite 5,6, 757 Pacific Hwy, Mount Kuring-Gai NSW 2080; 3 Department of Statistics and Epidemiology, Faculty of Health, Tabriz University of Medical Sciences, Tabriz, Iran.; 4 Indigenous & Global Health Research Group, Department of Medicine, University of Alberta, Edmonton, Canada.; 5 Department of Medicine, Faculty of Medicine and Dentistry, University of Alberta, Edmonton, Alberta, Canada.

**Keywords:** COPD, Antioxidant Vitamins, Fruit, Vegetables, Dietary Fiber, Fatty Acids

## Abstract

**Background:**

The relationship between dietary pattern and the risk of chronic obstructive pulmonary disease (COPD) has been described; however, the exclusive role of dietary factors remains controversial. Hence, we conducted this systematic meta-analysis to clarify the role of some nutrients and antioxidant vitamins in the risk of COPD.

**Materials and Methods:**

PubMed, Embase, and Scopus databases were searched for studies evaluating the associations between COPD outcome measures, symptoms, and mortality, and intake of fruits and vegetables, fiber, fish, n-3 or n-6 fatty acids, and antioxidant vitamins in adults. The random-effect model meta-analyses were used to pool the results.

**Results:**

Ten cohort, six case-control, and 20 cross-sectional studies were identified. The pooled relative risks (RRs) of the COPD and confidence intervals (CIs) for the highest intake group compared with the lowest intake group were 0.74 (95% CI: 0.65–0.85) for fruit, 0.65 (95% CI: 0.55–0.78) for dietary fiber, 0.71 (95% CI: 0.58–0.85) for fish, and 0.89 (95% CI: 0.76–0.99) for vitamin C. No association was observed between the risk of COPD and the intake of vegetables, n-3 fatty acids, vitamin E, and β-carotene; however, it was associated with n-6 fatty acids 1.06 (95% CI: 0.87–1.30).

**Conclusion:**

The results suggested that a higher intake of fruits, probably dietary fiber, and fish reduce the risk of COPD.

## INTRODUCTION

Chronic obstructive pulmonary disease (COPD) is a global public health problem and is a major cause of morbidity and mortality in developed and developing countries; according to estimations, it is the eighth cause of disability-adjusted life-years (DALYs) in 2016 in all age groups, and with an aging population, COPD prevalence is believed to increase (1–4). COPD is a preventable disease characterized by progressive airflow limitation, which only can be partially reversible. Smoking is the principal cause of COPD, but not all smokers develop the disease (2). Besides genetic factors, environmental exposures and dietary habits have been suggested as etiological factors for the risk of COPD (5–7). Additionally, the increased oxidative burden, as a major source in the pathogenesis of COPD, plays a critical role in lung injury and airway remodeling. One of the primary treatment targets of COPD is an improvement in the quality of life. In this regard, evidence highlights the importance of dietary modifications as antioxidant sources in the prevention and management of COPD (8, 9).

Several studies have suggested that specific foods and dietary supplements may be beneficial in COPD prevention and management (10, 11). Specific antioxidants (e.g., vitamins C and E), as well as foods rich in antioxidants (e.g., fruit and vegetables), appear to modulate lung function positively, airway damage, and COPD development and symptoms (12, 13). A recent review reported that a high intake of dietary fiber is associated with reduced COPD risk (14). In addition, fish and fatty acid consumption have both been directly correlated with respiratory symptoms of COPD (15). Omega-3 polyunsaturated fatty acids (n-3 PUFAs), including eicosapentaenoic acid (EPA) and docosahexaenoic acid (DHA), appear to have anti-inflammatory effects; however, there is contradictory evidence regarding the inverse association between intake of n-3 PUFAs and the risk of COPD and mortality (16). These findings have not yet been quantified in a comprehensive review or pooled using meta-analysis techniques. A recent review reviewed randomized controlled trials (RCTs) on the relationship between dietary pattern (nutritional supplementation) and the risk of COPD, in which no relationship was found between these two factors (17, 18). Thus, this study systematically investigated the association between some nutrients, including fruits, vegetables, fatty acids, and the antioxidant vitamins and the risk of COPD.

## MATERIALS AND METHODS

Both the systematic review and meta-analysis were conducted according to the Preferred Reporting Items for Systematic Reviews and Meta-Analyses (PRISMA) guidelines for reporting the current study (19).

### Search strategy and eligibility criteria

We searched PubMed (Medline), Embase, and Scopus databases for studies published in English from January 1990 to November 2018 on the associations between COPD outcomes and the intake of fish, fruits and vegetables, fiber, fatty acids (n-3 and n-6), and antioxidant vitamins (C, E, and β-carotene) in adults. Additional studies were identified by searching the reference list of the retrieved articles and the Science Citation Index manually.

**Figure 1.**
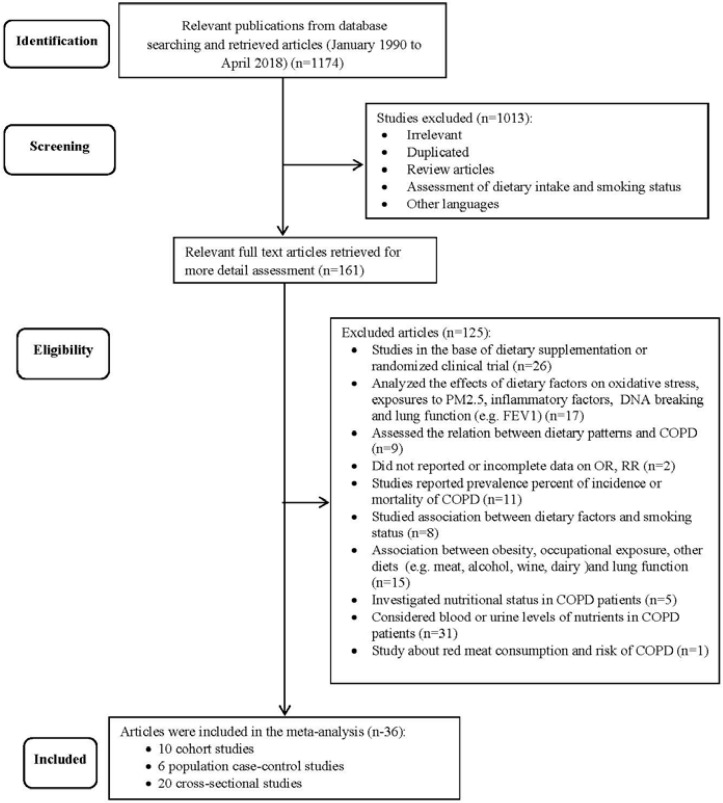
Study selection stages.

The following medical subject heading (MeSH) terms and/or keywords were used: “COPD”, “emphysema”, “chronic bronchitis” (as COPD outcomes), “COPD symptoms (including cough, phlegm, breathlessness, and dyspnea), or “COPD mortality”; and “diet”, “fish”, “fatty fish”, “fruit”, “citrus fruit”, “vegetables”, “dietary fiber”, “fatty acid”, “n-3 fatty acids”, “n-6 fatty acids”, “omega-3 fatty acid”, “omega-6 fatty acid”, “essential fatty acid”, “EFA (essential fatty acid)”, “polyunsaturated fatty acid”, “docosahexaenoic acid”, “eicosapentaenoic acid”, “fish oil”, “vitamin E”, “vitamin C”, “β-carotene”, “α-tocopherol”, and “ascorbic acid”. After retrieving the articles, prospective, case-control, cross-sectional, irrelevant, and duplicate studies, except for the most relevant articles were excluded. The extracted studies were independently inspected by two authors (ESR and MPM). Discrepancies were resolved by consensus, or, if needed, by arbitration from a senior author. We read the abstracts of all the remaining studies to exclude relevant articles. Studies that (i) only investigated the association between dietary supplementation among COPD patients, (ii) reported an association between blood levels of nutrients and risk of COPD, (iii) conducted diet pattern analyses, (iv) provided no data on relative risks (RRs) and or odds ratios (ORs), (v) reported duplicated data from another study, and (vi) provided no contrast between intake categories (Fig. 1) were also excluded. The full texts of the remaining articles were reviewed to determine the inclusion criteria and check the reported RR, OR, confidence intervals (CIs), or the required data to compute the COPD outcomes and symptoms to compare the high and low intake categories. The Critical Appraisal Skills Programme (CASP) was used to critically appraise the included observational studies (20).

### Data extraction

The following information was extracted from the included studies: publication date, type of study, author’s name, country, sample size, year of enrollment, participants’ characteristics, food items, dietary assessment methods, year of study, follow-up duration, potential confounding factors that accounted for COPD outcomes and symptoms, and smoking status.

### Data analysis and statistical methods

To conduct the meta-analysis, OR estimates from case-control and cross-sectional studies, and the risk or rate ratios from cohort studies, were assumed to be valid estimates of RR. RR estimates were pooled using the DerSimonian and Laird method through a weighted average of the log RR and considering random-effects. Heterogeneity for each pooled estimate was assessed using the Cochran’s Q-test. Heterogeneity was considered as P ≤ 0.1 for the Q statistic and regarded significant when I2 was >50%. Both the Begg’s rank correlation test and Egger’s regression model were used for assessing the publication bias. Analyses were conducted using Stata 13 software (Stata Corp LP, College Station, TX).

## RESULTS

The characteristics of the ten cohort studies (15, 21–29), six case-control studies (30–35), and 20 cross-sectional studies (36–55) included in the meta-analysis of the associations between dietary factors and the risk of COPD are shown in Table 1. Therefore, 36 publications were assessed in detail (Fig. 1).

**Table 1.** Characteristics of studies included in the meta-analysis of the association between dietary factors and COPD

Author name, year	Country	Sample Size	Year of Enrollment	Follow up (y)	Sex	Age(y)	Food items studies	Dietary evaluation	Contrast	Adjustment
**Cohort studies**										
Miedema et al., 1993(22)	Netherlands	793	1960	25	M	40–59	Fruit and fatty acids	Cross-check dietary method	Quartile 4 compared with quartile 1	Age, smoking, BMI, energy intake
Carey et al., 1998 (23)	United Kingdom	2,171	1984–1985	7	M/F	18–73	Fresh fruit	FFQ	4.5–5 times/d compared with never	Region, social class, smoking
Tabak et al., 1998 (21)	Seven Countries (Finland, Italy, Greece, former Yugoslavia, Japan, United States and Serbia)	12,763	1958–1964	25	M	40–59	Fish, n-3, n-6, EPA, DHA fatty acids, total fruit and vegetables, citrus fruit, other fruit, flavonoids, vitamins C, E and β -carotene	1, 4, and 7 day records from 16 different cohorts	The log of 10% of the mean of each food item intake	Energy intake, BMI
Walda et al., 2002 (24)	Finland, Italy and Netherlands	2,917	1958–1964	20	M	50–69	Fruit, vegetables, fish, vitamins C, E and β -carotene	Cross-check method	Highest vs. the lowest tertile	Country, age, smoking
Butler et al., 2004 (25)	Singapore	63,257	1993–1998	5	M/F	45–74	Fruit, Non-starchy vegetables, fish, soy, isoflavonoids, vitamins C, E and β-carotene	FFQ	Highest vs. the lowest quartile or tertile	Age, sex, ethnicity, energy intake, smoking
Varraso et al., 2010 (26)	USA	111,580	1984–2000	16	M/F	40–74	Total fiber, cereals fiber, fruit fiber, vegetable fiber	FFQ	Highest vs. the lowest quintile	Age, sex, smoking, energy intake, BMI, US region, physician visits, physical activity, diabetes, intakes of omega-3 and cured meat,
Varraso et al., 2015 (15)	USA	120,175	1984–1998		M/F	F: 30–55 M: 40–75	Fish, n-3, n-6, EPA, DHA fatty acids,	FFQ	<1 vs ≥4 serving/wk	Age, smoking, race-ethnicity, physician visit, US region, educational, menopausal status, BMI, physical activity, multivitamin use, and energy intake, and modified prudent and Western dietary patterns.
Joshi et al., 2015 (27)	Korea	7106	2001–2006	6	M/F	40–69	vitamins C, E and β -carotene	FFQ	Highest vs. the lowest quartile	age, sex, marriage status, BMI, history of asthma and tuberculosis, energy, and smoking.
Kaluza et al., 2017 (28)	Sweden	44335	1998–2012	13.2	M	45–79	Fruit, vegetables	FFQ	Highest vs. the lowest quintile	age, education, BMI, physical activity, smoking(status and pack-years), energy intake, alcohol consumption and modified recommended food score and non-recommended food score
Kaluza et al., 2018 (29)	Sweden	45058	1998–2012		M	45–79	Dietary fiber	FFQ	FFQ	age, education, BMI, physical activity, smoking(status and pack-years), energy intake, alcohol consumption
**Population case-control studies**										
Chen et al., 2001(34)	United Kingdom	364/374	1995	-	M/F	25–65	vitamins C, E and β-carotene	FFQ	Highest vs. the lowest quartile	age, sex, BMI, working status, energy intake, cotinine levels, and smoking pack years)
Watson et al., 2002 (30)	United Kingdom	150/116		-	M/F	>45	Fruit and vegetables	Validated FFQ	Highest vs. the lowest tertile	Age, BMI, vegetable intake
Celik et al., 2006 (31)	Turkey	40/36	2003–2004	-	M	Mean: cases:57.73 controls: 55.25	Fruit and vegetables, n-3 and n-6 fatty acids	Arizona FFQ (AFFQ)	>3times/day compared with never/rarely intake	Age, sex, smoking
Hirayama et al., 2009 (32)	Japan	278/340	2006	-	M/F	50–75	Fruit and vegetables, fiber, n-3 and n-6 fatty acids, vitamin C and β -carotene	Validated FFQ	≥7 times/d compared with almost never	Age, gender, BMI, education level, alcohol intake, smoking, physical activity, daily intake of red meat, chicken and fresh fish.
Hirayama et al., 2010 (33)	Japan	278/340	2006	-	M/F	50–75	Fatty acids, isoflavones(genistein and daidzein)	Validated FFQ	Highest vs. the lowest quartile	Age, gender, BMI, education level, alcohol intake, smoking, physical activity, daily intake of red meat
Lin et al., 2010 (35)	Taiwan	34/43	2005–2006	-	M/F	≥50	Fruit, vegetables, vitamins C, E and total carotenoids	FFQ	Fruit and vegetables: frequency/mo; Vitamins: mg/d	Age, sex, BMI, smoking, carbohydrate intake, protein intake
**Cross-sectional studies**										
Schwartz et al., 1990 (37)	USA	9,074	1976– 1980(NHANES II)	-	M/F	≥30	Fish and vitamin C	FFQ and 24hr recall	High vs. low intake	Age, race, sex, smoking (pack years), total calories
Strachan et al., 1991(38)	United Kingdom	1,357current smoker/1,502non-smokers	1984–1985	-	M/F	18–69	Fresh fruit and fruit juice	Validated FFQ	> once/d compared with never	Age, sex, height, smoking, region, household socioeconomic group
Shahar et al., 1994 (39)	USA	8,960	1986–1989		M/F	45–64	Fish and n-3 fatty acids	FFQ	Highest vs. the lowest quartile	Age, sex, race, height, weight, energy intake, educational levels, smoking (status and pack years)
Schwartz et al., 1994 (40)	USA	2,526	1971–1975 (NHANES I)	-	M/F	30–70	Fish	FFQ and 24hr dietary recall	Fish: Portion/wk Vit C: Highest vs. the lowest tertile	Age, sex, height, smoking, race, employment
Sharp et al., 1994 (41)	USA	6,346	1965–1968	-	M	45–68	Fish	FFQ	High(≥2times/wk) vs. low<2times/wk) fish intake	Age, height, smoking, energy intake, education level, body weight.
Britton et al., 1995 (42)	United Kingdom,	2,633	January to May 1991	-	M/F	18–70	Vitamins C and E	FFQ	Vit C: >40.2 mg from 99.2(mean)and Vit E: >2.2 mg from 6.2	Age, sex, height, mean allergen skin wheal diameter, smoking (status and pack years)
Dow et al., 1996 (43)	United Kingdom	178	June 1991–March 1992	-	M/F	70–96	Vitamins C and E	FFQ	Increase of each mg/d for vitamin E	Age, sex, height, smoking, energy intake, vitamin C intake
Rautalahti et al., 1997 (44)	Finland	7,286	1985– 1988	-	M	50–69	Vitamins E and β - carotene	FFQ	High vs. low frequency intake	
Grievink et al., 1998 (45)	Netherlands	6,555	1994– 1995	-	M/F	20– 59	Vitamins C, E and β -carotene	FFQ	90^th^ vs. 10^th^ percentile of antioxidant intake	Age, sex, energy intake, smoking (pack years), antioxidant intake
La Vecchia et al., 1998 (46)	Italy	46,693	1993	**-**	M/F	≥15	Vegetables	FFQ	Highest vs. the lowest tertile	Age, sex, alcohol intake, smoking, education.
Fluge et al., 1998 (47)	Norway	4,300	1991	-	M/F	20–44	Fish	FFQ	>1 time/wk compared with <1 time/wk	Age, sex, BMI, occupation, smoking
Hu et al., 1998 (48)	China	3,085	1989	-	M/F	35– 64	Vitamin C	3-day weighed household food record	Increase of 100 mg/d from mean (151.1) daily vitamin C intake	Age, sex, height, weight, education, smoking
Tabak et al., 1999 (49)	Finland, Italy and Netherlands	Finland: 1,248, Italy: 1,386 Netherland: 691	1960s	-	M	40–59	Fish	Cross-check dietary method	Highest vs. the lowest quartile	Height, age, smoking, BMI, alcohol intake, and energy intake
Hu et al., 2000 (50)	USA,	18,162	1988– 1994	-	M/F	≥17	Vitamins C, E and β-carotene	24hr dietary recall	Increase of 111 mg/d from mean (151.1) vitamin C and >9.1 α-TE/d vitamin E intake	Age, sex, height, age^2,^ race, BMI, income, smoking, total caloric and fat intake
Tabak et al., 2001 (51)	Netherlands	13,651	1994–1997		M/F	20–59	Fruit and vegetables,	FFQ	Highest vs. the lowest quartile	Age, sex, height, smoking, BMI, energy intake
Kelly et al., 2003 (52)	UK (Scotland)	1,146	1995		M/F	16–64	Fruit, vegetables, and fish	Questionnaire	At least once/d compared with never or rarely intake	Age, sex, height, age^2^, height^2^, smoking, social class, activity level.
Kan et al., 2008 (53)	USA	11,897	1987–1989		M/F	44– 66	Fiber	FFQ	Highest vs. the lowest quintiles	Age, sex, height, height^2^, study center, ethnicity, smoking (status and pack years), BMI, occupation, education, diabetes status, traffic, energy intake, glycemic index, micronutrients from food and supplements, cured meat, and fiber intake
McKeever et al., 2008 (36)	Netherlands	13,820	1994–1997	-	M/F	20–59	n-3, and n-6 fatty acids	FFQ	Highest vs. the lowest quintiles	Age, age^2^, sex, smoking, height, energy intake, vitamin C, BMI, education.
Vukovic et al., 2010 (54)	Serbia	14,522	2006	-	M/F	>20	Fruit and vegetables	Questionnaire	Every da intake compared with Less than every day	Age, sex, education, settlement type
Park et al., 2016 (55)	Korea	3,283	2012	-	M/F	≥40	Vtamin C	FFQ	Highest vs. the lowest quartile	age, sex, Univariate and multivariate analysis

Included studies had been conducted in different geographical areas as follows: eight studies in Asia (25, 27, 31–33, 35, 48, 55), 19 studies in Europe (22–24, 28–30, 34, 36, 38, 42, 43, 44–47, 49, 51, 52, 54), eight studies in the United States (15, 26, 37, 39–41, 50, 53) and one study in seven countries in Asia, Europe, and North America (Table 1). The characteristics of the included studies are displayed in Table 1. Most studies had been adjusted for age, sex, BMI, energy intake, smoking status, and social history.

To assess usual dietary intake, 28 studies had used food frequency questionnaires (FFQ), of which two studies had used FFQ and a 24–hour recall, three studies had used the crosscheck dietary method, one study had assessed with only 24-hour recall, and one study had used weighted household food records.

In the final analyses, the following number of studies had reported data for the associations between the risk of COPD (outcomes and symptoms) or mortality and the following dietary factors: seven for fruits (22, 25, 28, 30, 32, 54, 56), six for vegetables (25, 28, 30, 32, 46, 54), three for fruits & vegetables (28, 31, 35), five for fiber intake (25, 26, 29, 32, 53), six for n-3 fatty acids (15, 22, 32, 33, 36, 39), four for n-6 fatty acids (15, 32, 33, 36), two for linolenic acids (32, 36), three for linoleic acid (22, 32, 36), seven for fish (15, 21, 24, 37, 39, 47, 52), six for vitamin C (27, 32, 34, 35, 37, 55), five for vitamin E (27, 34, 35, 44, 45), and six for β-carotene (27, 32, 34, 35, 44, 45).

**Figure 2.**
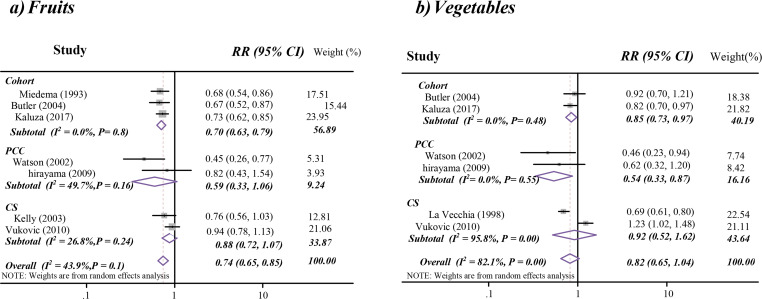

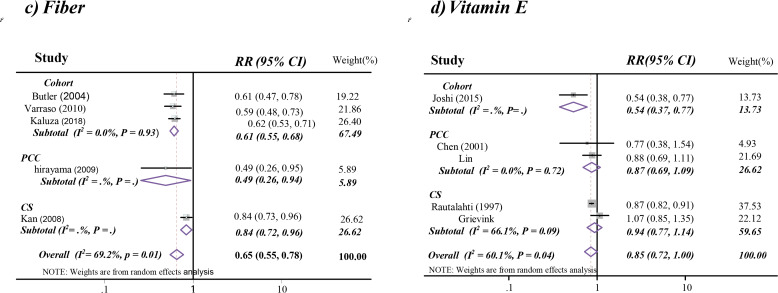
Forest plots of the association between intake of fruit (a), vegetables (b), dietary fiber (c), and vitamin E (d) and COPD outcomes and symptom.

The pooled analysis of seven studies showed a 26% reduction in the risk of COPD (symptom and outcomes) that was significantly associated with high fruit intake without heterogeneity (Table 2 and Fig. 2a). In this regard, the results of sensitivity analysis of the study design for three cohort studies (22, 25, 28) were: RR=0.71, 95% CI: 0.63– 0.79; I2: 0.0%, heterogeneity P-value: 0.8; for two population case-control studies (30, 32) were: RR= 0.59, 95% CI: 0.33– 1.06; I2: 50%, heterogeneity P-value: 0.2; and for two cross-sectional studies (54, 56) were: RR= 0.88, 95% CI: 0.72– 1.07; I2: 27%, heterogeneity P-value: 0.2. In addition, in the subgroup meta-analysis for five included studies reporting an association between fruits intake and COPD outcomes (22, 28, 30, 32, 54) or three studies regarding symptoms (25, 32, 56), there were no changes in the pooled effect estimates as displayed in Table 2. Meta-analysis on the association between COPD mortality and higher fruit consumption also showed a significant reduction in the risk of COPD mortality (RR=0.53; 95% CI: 0.39–0.74; I2: 0.0%; heterogeneity P-value: 0.65) (Table 2).

**Table 2.** Meta-analysis of the association between dietary intake and the risk of COPD

	Pooled estimate RR (95%CI)^*^	P heterogeneity	*I^2^*	No of studies
**Fruit**				

**COPD symptoms and outcomes**	0.74 (0.65–0.85)	0.1	44%	7
**COPD Outcomes**	0.74 (0.62–0.90)	0.04	59%	5
**COPD symptoms**	0.73 (0.61–0.88)	0.6	0.0%	3
**COPD mortality**	0.53 (0.39–0.74)	0.7	0.0%	2

**Vegetables**				

**COPD Symptoms & Outcomes**	0.82 (0.65–1.04)	<0.001	82%	6
**COPD Outcomes**	0.79 (0.60–1.05)	<0.01	85%	5
**COPD Symptoms**	0.71 (0.39–1.3)	0.1	72%	2
**COPD mortality**	1.1 (0.93–1.2)	0.7	0.0%	2

**Total fruit and vegetables**				

**COPD Outcomes**	0.80 (0.60–1.1)	<0.001	85%	3

**Dietary fiber**				

**COPD Symptoms & Outcomes**	0.65 (0.55–0.78)	0.01	69%	5
**COPD Outcomes**	0.65 (0.49–0.91)	0.01	77%	3

**n-3 fatty acids**				

**COPD Symptoms & Outcomes**	0.86 (0.66–1.11)	0.002	74%	6
**COPD Outcomes**	0.83 (0.67–1.03)	0.004	72%	6
**COPD Symptoms**	1.2 (1.0–1.4)	0.7	0.0%	2

**n-6 fatty acids**				

**COPD Symptoms & Outcomes**	1.06 (0.87–1.30)	0.14	45%	4
**COPD Outcomes**	1.04 (0.76–1.41)	0.1	55%	4
**COPD symptoms**	0.83 (0.38–1.86)	0.03	80%	2

**Fish**				

**COPD Symptoms & Outcomes**	0.71 (0.58–0.85)	0.002	76%	5
**COPD Outcomes**	0.65 (0.52–0.82)	0.03	72%	3
**COPD Symptoms**	0.81 (0.71–0.92)	0.7	0.0%	2
**COPD mortality**	0.96 (0.92–1.0)	0.8	0.0%	2

**Vitamin E**				

**COPD Symptoms & Outcomes**	0.85 (0.72–1.0)	0.04	60%	5
**COPD Outcomes**	0.79 (0.66–0.96)	0.1	56%	4
**COPD Symptoms**	0.93 (0.76–1.2)	0.1	67%	2
**COPD mortality**	0.95 (0.86–1.1)	0.4	0.0%	2

**β-carotene**				

**COPD Symptoms & Outcomes**	0.91 (0.78–1.05)	<0.001	95.4%	6
**COPD Outcomes**	0.92 (0.76–1.1)	<0.001	92%	5
**COPD Symptoms**	0.88 (0.63–1.3)	0.001	91%	2
**COPD mortality**	0.63 (0.35–1.1)	0.4	0.0%	2

**Vitamin C**				

**COPD Outcomes**	0.89(0.76–0.99)	0.002	73%	6
**COPD mortality**	0.55 (0.3–1.0)	0.9	0.0%	2

* The overall pooled risk ratio (RR) of COPD risk and the confidence intervals (CIs) for the groups with the highest versus the lowest levels of intake

** Population case-control

When the highest intake category of vegetables was compared with the lowest intake category, the pooled RR was null for the associated COPD symptoms and outcomes (Table 2). Following stratification by study design as a sensitivity analysis, the pooled RRs for two cohort studies (25, 28) were 0.85, 95%CI: 0.74–0.97; I2: 0.0%; heterogeneity P-value: 0.5; for two population case-control studies (30, 32) were 0.54, 95%CI: 0.33–0.87; I2: 0.0%; heterogeneity P-value: 0.6 and for two case-control studies (46, 54) were 0.92, 95%CI: 0.52–1.62; I2: 96%; heterogeneity P-value: <0.001. No association was found in two studies that had investigated vegetable intake and the risk of COPD mortality (21, 24). In addition, there was no association between COPD risk and the total intake of fruits and vegetables (Table 2).

A meta-analysis of the relationship between dietary fiber consumption and COPD risk (symptoms and outcomes), showed a 35% reduction in COPD risk (Table 2 and Fig. 2c). In this regard, stratification by study type showed a 39% reduction in the COPD risk in three cohort studies (RR=0.61; 95% CI: 0.54–0.68; I2: 0.0%; heterogeneity P-value: 0.9) (25, 26, 29). The pooled analysis for the association between COPD outcomes and dietary fiber intake also indicated a significant reduction in outcomes (RR=0.65; 95% CI: 0.49–0.91; I2: 77%; heterogeneity P-value: 0.01) (26, 29, 32, 53).

Among six studies investigating n-3 fatty acids, there were no associations between their high intake and risk of either COPD outcomes alone or both COPD symptoms and outcomes (Table 2). Similarly, the subgroup analysis according to study design exhibited no relationship between these factors as follows: two cohort studies (RR=1.00; 95% CI: 0.79–1.27; I2: 41%; heterogeneity P-value: 0.2) (15, 22); two population case-control studies (RR=0.79; 95% CI: 0.49–1.27; I2: 0.0%; heterogeneity P-value: 0.7) (32, 33) and two cross-sectional studies ((RR=0.74; 95% CI: 0.39–1.38; I2: 90%; heterogeneity P-value: 0.001) (36, 39). The pooled analysis of n-3 fatty acids and COPD symptoms indicated a positive relationship (Table 2) (33, 36). Also, there was an association between Linolenic acids intake and COPD risk (RR=1.02; 95% CI: 0.74–1.41; I2: 0.0%; heterogeneity P-value: 0.6). Of four studies investigating n-6 fatty acids, there were positive associations between their intake and risk of COPD (Table 2). Studies that specifically had investigated linoleic acids also displayed positive associations with COPD risk (RR=1.25; 95% CI: 0.88–1.77; I2: 52.5%; heterogeneity P-value: 0.1).

The pooled analysis of three studies on fish intake showed a 35% reduction in the risk of COPD (RR=0.65; 95% CI: 0.52–0.82; I2: 72%; heterogeneity P-value: 0.03). There was also a decrease in the risk of COPD by analysis of the included studies on both symptoms and outcomes (RR=0.71; 95% CI: 0.58–0.85; I2: 76%; heterogeneity P-value: 0.002); a considerable heterogeneity among these studies was observed. High fish intake was not associated with the risk of COPD mortality (21, 24); however, a significant decrease (19%) in the risk of COPD had been reported considering symptoms alone (RR=0.81; 95% CI: 0.71–0.92; I2: 0.0%; heterogeneity P-value: 0.73) (Table 2) (47, 52).

The pooled estimation was not significant regarding studies on the association of high intakes of vitamin E and risk of both COPD symptoms and outcomes (RR=0.85; 95% CI: 0.72–1.0; I2: 60%; heterogeneity P-value: 0.04) (Table 2 and Fig. 2d). The detailed analysis based on COPD symptoms, outcomes, or mortality is shown in Table 2. High intake of β-carotene was not associated with a risk of COPD in either the pooled analysis (Table 2) or after stratification by the study types, RR=0.99; 95% CI: 0.99–1.0; I2: 0.0%; heterogeneity P-value: 0.9 for population case-control studies (32, 34, 35) and RR=0.83; 95% CI: 0.74–0.93; I2: 0.0%; heterogeneity P-value: <0.001 for cross-sectional studies (44, 45). In addition, a high intake of vitamin C was associated with a reduced risk of COPD according to six studies, yet with a substantial heterogeneity (Table 2). The similar results were identified in subgroup analysis based on study design, RR=0.81; 95% CI: 0.55–1.17; I2: 51%; heterogeneity P-value: 0.1 for three population case-control studies (32, 34, 35) and RR=0.86; 95% CI: 0.76–0.99; I2: 85%; heterogeneity P-value: 0.01 for cross-sectional studies (37, 55).

The distribution of the funnel plot and Egger’s regression analysis did not show asymmetry. The cumulative analyses showed consistency of the findings over time (Figures not shown).

## DISCUSSION

There has been growing interest in the effects of foods and nutrients with antioxidant or anti-inflammatory properties on lung function or COPD symptoms (33). These investigations are mainly epidemiologic studies with inconsistent results.

This study was intended to systematically review all epidemiologic evidence related to the association between some antioxidant and anti-inflammatory dietary factors and COPD. The results showed that ample consumption of fruit significantly reduced the risk of COPD outcomes and symptoms. In addition, subgroup analyses by study design, as a sensitivity analysis, similarly supported the results obtained through a meta-analysis of longitudinal studies. Fiber intake reduced the risk of COPD outcomes and symptoms according to cohort studies, whereas n-3 and n-6 fatty acids intakes were not associated with the risk of COPD. The findings also indicated that high vitamin E intake significantly reduced the risk of COPD outcomes and symptoms.

According to epidemiological studies, diets rich in fruits, vegetables, and vitamins protect against chronic diseases, such as cardiovascular disease and cancer. Fruits and vegetables are rich in several types of vitamins and bioactive components (57). It has been reported that increased oxidative stress is a potential pathogenic factor for COPD (58). Therefore, it is suggested that the negative association between fruits and vegetables intake and COPD-related outcomes might be partly due to the antioxidant properties of these nutrients, such as vitamin C, flavonoids, and carotenoids (26, 59). Previous studies have supported the protective effect of flavonoids on COPD symptoms (25, 60). These compounds may protect against oxidant-mediated damage that leads to COPD (27). Although these antioxidants can be found in almost all fruits and vegetables, fruits have higher levels of antioxidants (61). This difference suggests a possible explanation for the observation that the association between COPD and high fruit intake was stronger than high vegetable intake.

Notably, longitudinal studies support the negative association between fiber intake and COPD that can be related to anti-inflammatory properties of dietary fiber (26). In agreement with our findings, some prospective studies supported strong associations between long-term increased fruits and fiber intake and health conditions (62). Fiber intake is associated with lower levels of C-reactive protein and pro-inflammatory cytokines and higher levels of some anti-inflammatory cytokines, such as adiponectin (63, 64). Lignans, which are constituents of dietary fibers and whole grains, inhibit type I–IV allergic inflammation and pro-inflammatory enzymes (65); however, the exact mechanism underlying dietary anti-inflammatory activity of fiber is unclear (26).

A healthy lifestyle, and a healthy diet, in particular, might be another explanation for the observed association between fruits intake and COPD related outcomes (66). Given that significant results were observed in longitudinal and cross-sectional studies, and also the included studies were mostly adjusted for several indicators of a healthy lifestyle (e.g., smoking, alcohol consumption, physical activity, and energy intake), the findings suggested that factors associated with a healthy lifestyle may not substantially affect the relationship between fruits, vegetables, and fiber intake and the risk of COPD. Another underlying issue regarding the association observed in this study might be the confounding effects of smoking. It has reported that smokers have lower intakes of fruit, vegetables, and whole grain products; on the other hand, they higher need vitamin C and β-carotene (66). The extent to which residual confounding factors influenced the results might not be significant, as most of the included studies had reported adjusted effect sizes; however, it cannot be concluded that underlying factors related to a healthy diet or the residual confounding effect of smoking may affect the observed associations.

The findings showed that vitamin E reduced the risk of COPD symptoms; however, no associations were found in pooled analysis of both symptoms and outcomes. This antioxidant vitamin may protect the lungs from oxidative damage caused by smoking or air pollution. Vitamin E is a free radical scavenger found in tissue membrane, as well as intracellular and extracellular lung fluids. In extracellular lung fluid and lipid membranes, vitamin E converts oxygen radicals and lipid peroxyl radicals to less reactive forms (67). A negative relationship was also observed between the intake of vitamin C and the risk of COPD. In this study, the interaction between these vitamins had not been investigated, and their synergic effects had not been quantified. Additionally, considering the biological interaction between vitamins C and E, the extent to which the protective effect of vitamin E is related to that of vitamin C is unresolved (68).

The results of the meta-analysis did not support the association between the intake of unsaturated fatty acids (n-3 and n-6 fatty acids) and the risk of COPD. Observational studies have been conducted based on the hypothesis that n-6 fatty acids could stimulate the production of pro-inflammatory eicosanoids, while anti-inflammatory n-3 fatty acids might promote the metabolism of these molecules into less biologically active eicosanoids, such as leukotriene B5 (69).

The current study had some limitations. Some analyses were based on only two studies, and few prospective studies were included. Furthermore, the null findings suggest that the effect of any individual nutrient in reducing the risk of COPD may be too small to detect; however, when several nutrients are consumed together, the cumulative effect may be sufficient for detection (17). Although some articles were cross-sectional studies, but the cohort study, which included in the pooled analysis studies, exhibited logical outcomes. Another limitation of this meta-analysis was the heterogeneity identified in some of the pooled analyses, which may be attributed to the sample sizes, small number of the studies included, or the distribution of effective modifiers that were not investigated in the primary studies and could not be detected. Studying an overall dietary approach rather than specific foods or nutrients is effective in investigating the association between dietary patterns and diseases more comprehensively. However, this study revealed a strong negative association with taking fruits and the risk of COPD (70).

Our results support nutritional interventions encouraging taking fruits, and probably fish, and dietary fiber, which can reduce the risk of COPD outcomes and symptoms significantly.

## References

[1] GBD 2015 Disease and Injury Incidence and Prevalence Collaborators Global, regional, and national incidence, prevalence, and years lived with disability for 310 diseases and injuries, 1990–2015: a systematic analysis for the Global Burden of Disease Study 2015. Lancet 2016;388(10053):1545–1602. 27733282 10.1016/S0140-6736(16)31678-6 PMC5055577

[2] Mannino DM Buist AS Global burden of COPD: risk factors, prevalence, and future trends. Lancet 2007;370(9589):765–73. 17765526 10.1016/S0140-6736(07)61380-4

[3] Halbert RJ Natoli JL Gano A Badamgarav E Buist AS Mannino DM Global burden of COPD: systematic review and meta-analysis. Eur Respir J 2006;28(3):523–32. 16611654 10.1183/09031936.06.00124605

[4] GBD 2016 DALYs and HALE Collaborators Global, regional, and national disability-adjusted life-years (DALYs) for 333 diseases and injuries and healthy life expectancy (HALE) for 195 countries and territories, 1990–2016: a systematic analysis for the Global Burden of Disease Study 2016. Lancet 2017;390(10100):1260–1344. 28919118 10.1016/S0140-6736(17)32130-X PMC5605707

[5] Bruse S Moreau M Bromberg Y Jang JH Wang N Ha H Whole exome sequencing identifies novel candidate genes that modify chronic obstructive pulmonary disease susceptibility. Hum Genomics 2016;10:1. 26744305 10.1186/s40246-015-0058-7 PMC4705629

[6] Kim WJ Lee SD Candidate genes for COPD: current evidence and research. Int J Chron Obstruct Pulmon Dis 2015;10:2249–55. 26527870 10.2147/COPD.S80227 PMC4621177

[7] Yao H Rahman I Current concepts on oxidative/carbonyl stress, inflammation and epigenetics in pathogenesis of chronic obstructive pulmonary disease. Toxicol Appl Pharmacol 2011;254(2):72–85. 21296096 10.1016/j.taap.2009.10.022 PMC3107364

[8] Denny SI Thompson RL Margetts BM Dietary factors in the pathogenesis of asthma and chronic obstructive pulmonary disease. Curr Allergy Asthma Rep 2003;3(2):130–6. 12562552 10.1007/s11882-003-0025-6

[9] Brug J Schols A Mesters I Dietary change, nutrition education and chronic obstructive pulmonary disease. Patient Educ Couns 2004;52(3):249–57. 14998594 10.1016/S0738-3991(03)00099-5

[10] Hirayama F Lee AH Binns CW Dietary factors for chronic obstructive pulmonary disease: epidemiological evidence. Expert Rev Respir Med 2008;2(5):645–53. 20477299 10.1586/17476348.2.5.645

[11] van de Bool C Mattijssen-Verdonschot C van Melick PP Spruit MA Franssen FM Wouters EF Quality of dietary intake in relation to body composition in patients with chronic obstructive pulmonary disease eligible for pulmonary rehabilitation. Eur J Clin Nutr 2014;68(2):159–65. 24327123 10.1038/ejcn.2013.257

[12] Kurti SP Murphy JD Ferguson CS Brown KR Smith JR Harms CA Improved lung function following dietary antioxidant supplementation in exercise-induced asthmatics. Respir Physiol Neurobiol 2016;220:95–101. 26453914 10.1016/j.resp.2015.09.012

[13] Hong JY Lee CY Lee MG Kim YS Effects of dietary antioxidant vitamins on lung functions according to gender and smoking status in Korea: a population-based cross-sectional study. BMJ Open 2018;8(4):e020656. 10.1136/bmjopen-2017-020656 PMC5892775 29627816

[14] Fonseca Wald ELA van den Borst B Gosker HR Schols AMWJ Dietary fibre and fatty acids in chronic obstructive pulmonary disease risk and progression: a systematic review. Respirology 2014;19(2):176–184. 24372903 10.1111/resp.12229

[15] Varraso R Barr RG Willett WC Speizer FE Camargo CA Jr Fish intake and risk of chronic obstructive pulmonary disease in 2 large US cohorts. Am J Clin Nutr 2015;101(2):354–61. 25646333 10.3945/ajcn.114.094516 PMC4307205

[16] Pizzini A Lunger L Sonnweber T Weiss G Tancevski I The Role of Omega-3 Fatty Acids in the Setting of Coronary Artery Disease and COPD: A Review. Nutrients 2018;10(12). pii: E1864. 30513804 10.3390/nu10121864 PMC6316059

[17] Zheng PF Shu L Si CJ Zhang XY Yu XL Gao W Dietary Patterns and Chronic Obstructive Pulmonary Disease: A Meta-analysis. COPD 2016;13(4):515–22. 26678388 10.3109/15412555.2015.1098606

[18] Collins PF Elia M Stratton RJ Nutritional support and functional capacity in chronic obstructive pulmonary disease: a systematic review and meta-analysis. Respirology 2013;18(4):616–29. 23432923 10.1111/resp.12070

[19] Moher D Liberati A Tetzlaff J Altman DG PRISMA Group Preferred reporting items for systematic reviews and meta-analyses: the PRISMA statement Ann Intern Med 2009;151(4):264–9, W64. 19622511 10.7326/0003-4819-151-4-200908180-00135

[20] Weightman AL Mann MK Sander L Turley RL Questions to assist with the critical appraisal of an observational study eg cohort, case-control, crosssectional. A systematic approach to identifying the evidence, Project methodology 5. Cardiff: Information Services UWCM, 1 2004 http://hebw.uwcm.ac.uk/projectmethod/Project%20Methodology%205.pdf.

[21] Tabak C Feskens EJ Heederik D Kromhout D Menotti A Blackburn HW Fruit and fish consumption: a possible explanation for population differences in COPD mortality (The Seven Countries Study). Eur J Clin Nutr 1998;52(11):819–25. 9846595 10.1038/sj.ejcn.1600653

[22] Miedema I Feskens EJ Heederik D Kromhout D Dietary determinants of long-term incidence of chronic nonspecific lung diseases. The Zutphen Study. Am J Epidemiol 1993;138(1):37–45. 8333425 10.1093/oxfordjournals.aje.a116775

[23] Carey IM Strachan DP Cook DG Effects of changes in fresh fruit consumption on ventilatory function in healthy British adults. Am J Respir Crit Care Med 1998;158(3):728–33. 9730997 10.1164/ajrccm.158.3.9712065

[24] Walda IC Tabak C Smit HA Räsänen L Fidanza F Menotti A Diet and 20-year chronic obstructive pulmonary disease mortality in middle-aged men from three European countries. Eur J Clin Nutr 2002;56(7):638–43. 12080403 10.1038/sj.ejcn.1601370

[25] Butler LM Koh WP Lee HP Yu MC London SJ Dietary fiber and reduced cough with phlegm: a cohort study in Singapore. Am J Respir Crit Care Med 2004;170(3):279–87. 15117740 10.1164/rccm.200306-789OC

[26] Varraso R Willett WC Camargo CA Jr Prospective study of dietary fiber and risk of chronic obstructive pulmonary disease among US women and men. Am J Epidemiol 2010;171(7):776–84. 20172921 10.1093/aje/kwp455 PMC2877480

[27] Joshi P Kim WJ Lee SA The effect of dietary antioxidant on the COPD risk: the community-based KoGES (Ansan-Anseong) cohort. Int J Chron Obstruct Pulmon Dis 2015;10:2159–68. 26504380 10.2147/COPD.S91877 PMC4603710

[28] Kaluza J Larsson SC Orsini N Linden A Wolk A Fruit and vegetable consumption and risk of COPD: a prospective cohort study of men. Thorax 2017;72(6):500–509. 28228486 10.1136/thoraxjnl-2015-207851

[29] Kaluza J Harris H Wallin A Linden A Wolk A Dietary Fiber Intake and Risk of Chronic Obstructive Pulmonary Disease: A Prospective Cohort Study of Men. Epidemiology 2018;29(2):254–260. 28901975 10.1097/EDE.0000000000000750

[30] Watson L Margetts B Howarth P Dorward M Thompson R Little P The association between diet and chronic obstructive pulmonary disease in subjects selected from general practice. Eur Respir J 2002;20(2):313–8. 12212961 10.1183/09031936.02.00256402

[31] Celik F Topcu F Nutritional risk factors for the development of chronic obstructive pulmonary disease (COPD) in male smokers. Clin Nutr 2006;25(6):955–61. 16782241 10.1016/j.clnu.2006.04.006

[32] Hirayama F Lee AH Binns CW Zhao Y Hiramatsu T Tanikawa Y Do vegetables and fruits reduce the risk of chronic obstructive pulmonary disease? A case-control study in Japan. Prev Med 2009;49(2–3):184–9. 19555711 10.1016/j.ypmed.2009.06.010

[33] Hirayama F Lee AH Binns CW Hiramatsu N Mori M Nishimura K Dietary intake of isoflavones and polyunsaturated fatty acids associated with lung function, breathlessness and the prevalence of chronic obstructive pulmonary disease: possible protective effect of traditional Japanese diet. Mol Nutr Food Res 2010;54(7):909–17. 20112297 10.1002/mnfr.200900316

[34] Chen R Tunstall-Pedoe H Bolton-Smith C Hannah MK Morrison C Association of dietary antioxidants and waist circumference with pulmonary function and airway obstruction. Am J Epidemiol 2001;153(2):157–63. 11159161 10.1093/aje/153.2.157

[35] Lin YC Wu TC Chen PY Hsieh LY Yeh SL Comparison of plasma and intake levels of antioxidant nutrients in patients with chronic obstructive pulmonary disease and healthy people in Taiwan: a case-control study. Asia Pac J Clin Nutr 2010;19(3):393–401. 20805084

[36] McKeever TM Lewis SA Cassano PA Ocké M Burney P Britton J The relation between dietary intake of individual fatty acids, FEV1 and respiratory disease in Dutch adults. Thorax 2008;63(3):208–14. 17901161 10.1136/thx.2007.090399 PMC3979330

[37] Schwartz J Weiss ST Dietary factors and their relation to respiratory symptoms. The Second National Health and Nutrition Examination Survey. Am J Epidemiol 1990;132(1):67–76. 2356815 10.1093/oxfordjournals.aje.a115644

[38] Strachan DP Cox BD Erzinclioglu SW Walters DE Whichelow MJ Ventilatory function and winter fresh fruit consumption in a random sample of British adults. Thorax 1991;46(9):624–9. 1948789 10.1136/thx.46.9.624 PMC463343

[39] Shahar E Folsom AR Melnick SL Tockman MS Comstock GW Gennaro V Dietary n-3 polyunsaturated fatty acids and smoking-related chronic obstructive pulmonary disease. Atherosclerosis Risk in Communities Study Investigators. N Engl J Med 1994;331(4):228–33. 8015569 10.1056/NEJM199407283310403

[40] Schwartz J Weiss ST The relationship of dietary fish intake to level of pulmonary function in the first National Health and Nutrition Survey (NHANES I). Eur Respir J 1994;7(10):1821–4. 7828691 10.1183/09031936.94.07101821

[41] Sharp DS Rodriguez BL Shahar E Hwang LJ Burchfiel CM Fish consumption may limit the damage of smoking on the lung. Am J Respir Crit Care Med 1994;150(4):983–7. 7921474 10.1164/ajrccm.150.4.7921474

[42] Britton JR Pavord ID Richards KA Knox AJ Wisniewski AF Lewis SA Dietary antioxidant vitamin intake and lung function in the general population. Am J Respir Crit Care Med 1995;151(5):1383–7. 7735589 10.1164/ajrccm.151.5.7735589

[43] Dow L Tracey M Villar A Coggon D Margetts BM Campbell MJ Does dietary intake of vitamins C and E influence lung function in older people? Am J Respir Crit Care Med 1996;154(5):1401–4. 8912755 10.1164/ajrccm.154.5.8912755

[44] Rautalahti M Virtamo J Haukka J Heinonen OP Sundvall J Albanes D The effect of alpha-tocopherol and beta-carotene supplementation on COPD symptoms. Am J Respir Crit Care Med 1997;156(5):1447–52. 9372659 10.1164/ajrccm.156.5.96-11048

[45] Grievink L Smit HA Ocké MC van ’t Veer P Kromhout D Dietary intake of antioxidant (pro)-vitamins, respiratory symptoms and pulmonary function: the MORGEN study. Thorax 1998;53(3):166–71. 9659349 10.1136/thx.53.3.166 PMC1745167

[46] La Vecchia C Decarli A Pagano R Vegetable consumption and risk of chronic disease. Epidemiology 1998;9(2):208–10. 9504293

[47] Fluge O Omenaas E Eide GE Gulsvik A Fish consumption and respiratory symptoms among young adults in a Norwegian community. Eur Respir J 1998;12(2):336–40. 9727781 10.1183/09031936.98.12020336

[48] Hu G Zhang X Chen J Peto R Campbell TC Cassano PA Dietary vitamin C intake and lung function in rural China. Am J Epidemiol 1998;148(6):594–9. 9753014 10.1093/oxfordjournals.aje.a009685

[49] Tabak C Smit HA Räsänen L Fidanza F Menotti A Nissinen A Dietary factors and pulmonary function: a cross sectional study in middle aged men from three European countries. Thorax 1999;54(11):1021–6. 10525562 10.1136/thx.54.11.1021 PMC1745389

[50] Hu G Cassano PA Antioxidant nutrients and pulmonary function: the Third National Health and Nutrition Examination Survey (NHANES III). Am J Epidemiol 2000;151(10):975–81. 10853636 10.1093/oxfordjournals.aje.a010141

[51] Tabak C Smit HA Heederik D Ocké MC Kromhout D Diet and chronic obstructive pulmonary disease: independent beneficial effects of fruits, whole grains, and alcohol (the MORGEN study). Clin Exp Allergy 2001;31(5):747–55. 11422134 10.1046/j.1365-2222.2001.01064.x

[52] Kelly Y Sacker A Marmot M Nutrition and respiratory health in adults: findings from the health survey for Scotland. Eur Respir J 2003;21(4):664–71. 12762354 10.1183/09031936.03.00055702

[53] Kan H Stevens J Heiss G Rose KM London SJ Dietary fiber, lung function, and chronic obstructive pulmonary disease in the atherosclerosis risk in communities study. Am J Epidemiol 2008;167(5):570–8. 18063592 10.1093/aje/kwm343 PMC2377022

[54] Vukovic DS Nagorni-Obradovic LM Vukovic GM Lifestyle and perceived health in subjects with chronic bronchitis or emphysema: a cross-sectional study. BMC Public Health 2010;10:546. 20828414 10.1186/1471-2458-10-546 PMC2944375

[55] Park HJ Byun MK Kim HJ Kim JY Kim YI Yoo KH Dietary vitamin C intake protects against COPD: the Korea National Health and Nutrition Examination Survey in 2012. Int J Chron Obstruct Pulmon Dis 2016;11:2721–2728. 27843308 10.2147/COPD.S119448 PMC5098518

[56] Kelly FJ Vitamins and respiratory disease: antioxidant micronutrients in pulmonary health and disease. Proc Nutr Soc 2005;64(4):510–26. 16313695 10.1079/pns2005457

[57] Liu RH Health-promoting components of fruits and vegetables in the diet Adv Nutr 2013;4(3):384S–92S. 23674808 10.3945/an.112.003517 PMC3650511

[58] Boskabady MH Gholami Mahtaj L Lung inflammation changes and oxidative stress induced by cigarette smoke exposure in guinea pigs affected by Zataria multiflora and its constituent, carvacrol. BMC Complement Altern Med 2015;15:39. 25881210 10.1186/s12906-015-0574-y PMC4354995

[59] Zhai T Li S Hu W Li D Leng S Potential Micronutrients and Phytochemicals against the Pathogenesis of Chronic Obstructive Pulmonary Disease and Lung Cancer. Nutrients 2018;10(7). pii: E813. 10.3390/nu10070813 PMC6073117 29941777

[60] Tabak C Arts IC Smit HA Heederik D Kromhout D Chronic obstructive pulmonary disease and intake of catechins, flavonols, and flavones: the MORGEN Study. Am J Respir Crit Care Med 2001;164(1):61–4. 11435239 10.1164/ajrccm.164.1.2010025

[61] Van Duyn MA Pivonka E Overview of the health benefits of fruit and vegetable consumption for the dietetics professional: selected literature. J Am Diet Assoc 2000;100(12):1511–21. 11138444 10.1016/S0002-8223(00)00420-X

[62] Bertoia ML Mukamal KJ Cahill LE Hou T Ludwig DS Mozaffarian D Changes in Intake of Fruits and Vegetables and Weight Change in United States Men and Women Followed for Up to 24 Years: Analysis from Three Prospective Cohort Studies PLoS Med 2015;12(9):e1001878. 26394033 10.1371/journal.pmed.1001878 PMC4578962

[63] Ma Y Griffith JA Chasan-Taber L Olendzki BC Jackson E Stanek EJ 3rd Association between dietary fiber and serum C-reactive protein. Am J Clin Nutr 2006;83(4):760–6. 16600925 10.1093/ajcn/83.4.760 PMC1456807

[64] Sánchez D Miguel M Aleixandre A Dietary fiber, gut peptides, and adipocytokines. J Med Food 2012;15(3):223–30. 22181071 10.1089/jmf.2011.0072

[65] Lee JY Kim CJ Arctigenin, a phenylpropanoid dibenzylbutyrolactone lignan, inhibits type I–IV allergic inflammation and pro-inflammatory enzymes. Arch Pharm Res 2010;33(6):947–57. 20607501 10.1007/s12272-010-0619-1

[66] Sorli-Aguilar M Martin-Lujan F Flores-Mateo G Arija-Val V Basora-Gallisa J Sola-Alberich R RESET Study Group investigators Dietary patterns are associated with lung function among Spanish smokers without respiratory disease BMC Pulm Med 2016;16(1):162. 27884188 10.1186/s12890-016-0326-x PMC5123418

[67] Niki E Role of vitamin E as a lipid-soluble peroxyl radical scavenger: in vitro and in vivo evidence. Free Radic Biol Med 2014;66:3–12. 23557727 10.1016/j.freeradbiomed.2013.03.022

[68] Smit HA Grievink L Tabak C Dietary influences on chronic obstructive lung disease and asthma: a review of the epidemiological evidence. Proc Nutr Soc 1999;58(2):309–19. 10466172 10.1017/s0029665199000427

[69] de Silva PS Luben R Shrestha SS Khaw KT Hart AR Dietary arachidonic and oleic acid intake in ulcerative colitis etiology: a prospective cohort study using 7-day food diaries. Eur J Gastroenterol Hepatol 2014;26(1):11–8. 24216567 10.1097/MEG.0b013e328365c372

[70] Varraso R Fung TT Hu FB Willett W Camargo CA Prospective study of dietary patterns and chronic obstructive pulmonary disease among US men. Thorax 2007;62(9):786–91. 17504819 10.1136/thx.2006.074534 PMC2117325

